# Senescence of tumor cells induced by oxaliplatin increases the efficiency of a lipid A immunotherapy via the recruitment of neutrophils

**DOI:** 10.18632/oncotarget.2556

**Published:** 2014-10-16

**Authors:** Cédric Seignez, Amandine Martin, Claire-Emmanuelle Rollet, Cindy Racoeur, Alessandra Scagliarini, Jean-François Jeannin, Ali Bettaieb, Catherine Paul

**Affiliations:** ^1^ EPHE Cancer Immunotherapy Laboratory, EA7269 EPHE-University of burgundy, Dijon, F-21000, France

**Keywords:** senescence, colorectal cancer, immunotherapy associated chemotherapy, neutrophil

## Abstract

Management of advanced colorectal cancer is challenging due to the lack of efficient therapy. The lipid A, OM-174 exhibited antitumor activity in colorectal cancer. We explored the anticancer efficacy of this compound in rats bearing large colorectal tumors in combination with the platinum derivative drugs oxaliplatin and cisplatin. While each drug used alone exhibited partial antitumor activity, sequential treatment with oxaliplatin or cisplatin for one week followed by lipid A injections induced a great regression of colorectal tumors, with more than 95% of rats cured from their tumors. This potent antitumor efficacy of the combined treatments was correlated to the sequential induction of cellular senescence by oxaliplatin, and of apoptosis, mainly triggered by the lipid A. Moreover, a recruitment of Tumor-Associated Neutrophils with N1 phenotype as attested by the expression of inducible nitric oxide synthase was observed with combination of oxaliplatin and lipid A. Neutrophil recruitment within tumor microenvironment was due to oxaliplatin and lipid A-dependent release of neutrophil specific chemoattractants such as cxcl1 and 2. However the N1 phenotype is only dependent of lipid A treatment. These results suggest that the combination of chemotherapy with an immunotherapy is a promising approach to treat patients with advanced colorectal cancer.

## INTRODUCTION

Colorectal cancer is the second leading cause of death in Northern countries [1]. Despite significant developments in the treatment of this disease by using new cytotoxic chemotherapies and novel biological agents, it still causes considerable mortality particularly in patients with metastatic colorectal cancer (mCRC) [2]. Several drugs used as single agent or in various combinations are available to treat mCRC, including fluoropyrimidines (5FU, capecitabine), irinotecan, oxaliplatin, the vascular endothelial growth factor (VEGF) antibody bevacizumab, the epidermal growth factor receptor (EGFR) antibodies cetuximab and panitumumab, and others [3]. The antitumor efficacy of these drugs has been ascribed to their ability to block the growth of tumors through different mechanisms. They can induce apoptosis of tumor cells by the activation of series of cysteine proteases called caspases [4] or their senescence, a process leading to irreversible arrest of cell division. Cells can undergo senescence through three separate pathways [5]: (i) replicative senescence, induced through shortening of telomere; (ii) stress-induced premature senescence, triggered by cellular stress, such as elevated oxygen levels or cytotoxic agents leading to extensive DNA damage; and (iii) overexpression or hyperactivation of oncogenes, such as Ras, c-myc, or BRAF. The activation of any of these senescence pathways triggers a permanent arrest of growth of transformed cells and thus prevents carcinogenesis. Even though senescent cells cannot proliferate they are still metabolically active and release a broad variety of cytokines and chemokines that modify the microenvironment.

Despite the improvement of overall survival of patients with mCRC treated with these cytotoxic chemotherapies and biological agents, the development of resistances has been observed. Introduction of new treatment regimens is thus required to maximize therapeutic impact and tailor the toxicity profile in patients suffering from mCRC. Significant progress in understanding the tumor-induced immune suppression mechanisms allows the development of new immunological therapeutic strategies. To date the most effective immunological anti-tumor responses have been observed with active nonspecific approaches, such as Bacillus Calmette-Guérin (BCG) [6]. Moreover, recent data demonstrate that the immune infiltrates of the tumor such as T-lymphocytes are determinant for the outcome of patients bearing a solid cancer [7]. This suggests that patient prognosis can be estimated by the evaluation of the immune component of the tumor microenvironment. Furthermore, there is growing evidence that combination of conventional chemotherapy with immunomodulation strategies is of critical importance for efficient tumor eradication [8].

We have previously shown that an analog of lipid A, OM-174 (the active component of lipopolysaccharides) exerts, in experimental models, antitumor effect against different tumor types including colon, breast cancers and melanoma [9, 10]. In a model of peritoneal carcinomatosis induced in rats by intraperitoneal injection of syngeneic colon cancer cells, administration of lipid A induced complete regression of tumors and hemorrhagic ascites in 95% of cases [9]. This antitumor activity was associated with inflammatory cytokine secretion and inducible nitric oxide synthase (iNOS) activation [11]. However, lipid A, which safety and tolerance have been demonstrated in a phase I clinical trial in patients suffering from cancer [12], was not effective in rats bearing large tumors (21 days post-injection of cancer cells).

In the current study, we investigated the antitumor activity of platinum derivative oxaliplatin and cisplatin in combination with this analogue of lipid A in large peritoneal carcinomatosis. Moreover, we explored the mechanism of their action *in vitro* and *in vivo*, by studying their effects on apoptosis and senescence of tumor cells, and by assessing their ability to promote the recruitment of immune cells within tumors. We have shown that platinum derivatives induce tumor cell senescence which, when combined to lipid A, allows the recruitment of antitumoral neutrophils (N1), characterized by iNOS expression, within the tumors. Our findings indicate that combination of platinum derivative with lipid A is highly promising for the treatment of advanced colorectal tumors.

## RESULTS

### Association of platinum derivatives with an analogue of lipid A eradicated large colorectal tumors in syngeneic rats

**Figure 1:**
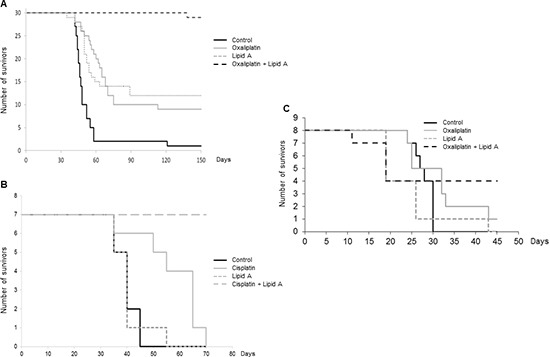
Oxaliplatin or cisplatin in combination with lipid A increased the lifespan of tumor-bearing rats or mice Tumor-bearing rats were treated by oxaliplatin/lipid A **(A)** or cisplatin/lipid A **(B)** combination. Twenty-one days after *i.p.* injection of 10^6^ PROb cells, rats were treated by oxaliplatin or cisplatin or saline solution (control). At twenty eight days, rats were treated with a lipid A or saline solution 3 times per week for 5 weeks. Figure A represented data from 3 independent experiments. Figure B was representative of 3 experiments with 7 animals per group. **(C)** Five days after i.p. injection of CT26 cells, mice were treated by oxaliplatin or saline solution (control). At ten days, mice were treated with a lipid A or saline solution five times, every five days.

BD IX rats bearing large syngeneic tumors due to PROb colon cancer cells (21 days after cell inoculation in rats) were randomized into 6 groups: control (vehicle), oxaliplatin or cisplatin (3 mg/kg), lipid A (1 mg/kg), and the combination of lipid A with each platinum derivative. Treatment with one dose of oxaliplatin or cisplatin was started at day 21 post-tumor cell injection. One week later, the rats were treated with lipid A three times per week, during five weeks. Figure 1A shows that the combination increased drastically the life span of rats since 95% of rats treated with oxaliplatin and lipid A were alive on day 150 and were cured of their affection (data not shown), while only 33–40% of rats treated with either lipid A or oxaliplatin and 3% of untreated rats were still alive at the same date. Furthermore, on day 70, all the rats of the control group or treated with cisplatin only were dead while all the rats treated with the combination cisplatin and lipid A were alive (Figure 1B). We also confirmed the antitumor efficacy of oxaliplatin and lipid A in the syngenic colon cancer CT26 mouse model. As shown in Figure 1C, 50% of mice were alive after 45 days of their treatment with oxaliplatin and lipid A while nearly all mice were died when left untreated or treated with drugs when taken alone. These results indicate that sequential treatment with platinum derivative and lipid A increased the life span of rats and mice bearing large tumors.

### Lipid A induced apoptosis of cancer cells which was increased by oxaliplatin

**Figure 2:**
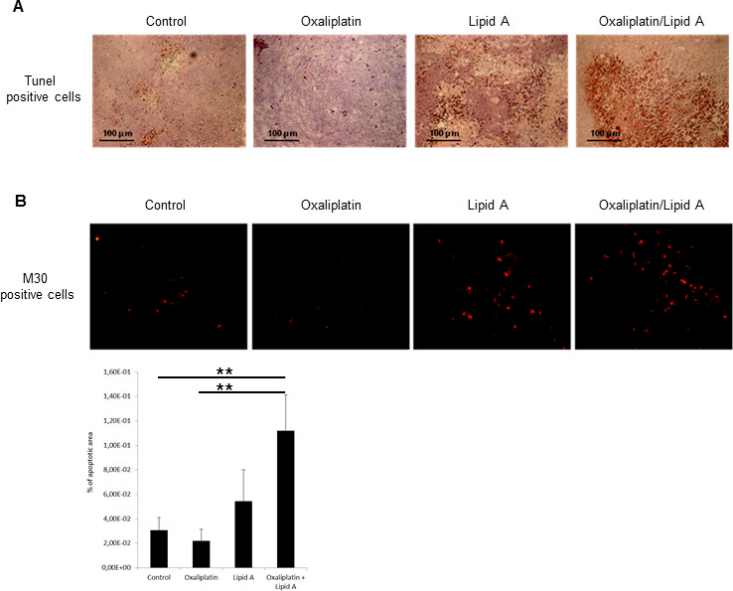
Lipid A induced apoptosis in tumor cells *in vivo*, increased by oxaliplatin Tumor-bearing rats were treated at day 21 with oxaliplatin or saline solution (control). At day 28, rats were treated with lipid A or saline solutions. Tumors were harvested at day 29. **(A)** Apoptosis in tumors was detected by TUNEL staining (brown staining, upper panel). **(B)** Apoptosis in tumor cells was detected by M30 immunostaining (red staining, lower panel). Images are representative of 3 independent experiments with 3 animals per group. Percentage of apoptotic area on slides was represented in the lower graph. Significant differences were determined by an Anova followed by a Bonferroni test.,**p < 0.01.

We have explored the mechanisms involved in the antitumor efficacy of platinum derivative/lipid A combination. We analyzed the ability of lipid A and oxaliplatin to induce apoptosis in tumor (TUNEL staining) and more precisely in tumor cells (M30 staining) (Figure 2A and B). The lipid A induced apoptosis in tumors after 24h time period treatment of rats. Oxaliplatin had little effect on this type of cell death induction but increased the rate of apoptosis induced by lipid A. Our results also indicated that triggering and increasing of apoptosis by lipid A and oxaliplatin, respectively, were correlated to caspase-3 activation as attested by the elevated rate of M30, the caspase 3-dependent cleaved form of cytokeratin 18.

### Oxaliplatin induced cellular senescence in colon cancer cell lines

**Figure 3:**
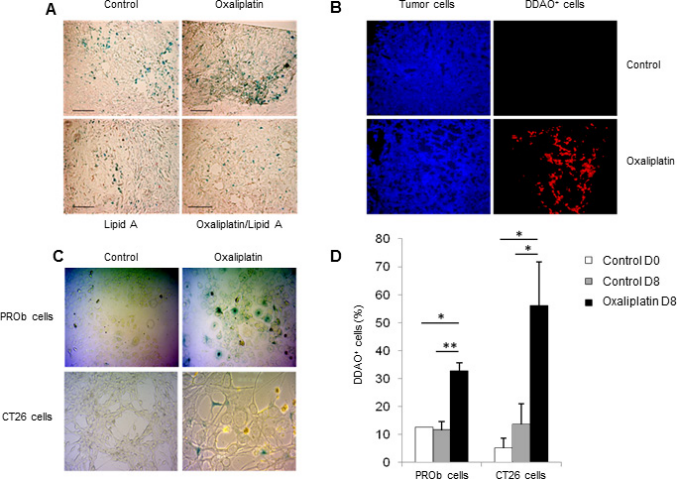
Oxaliplatin induced senescence of tumor cells *in vivo* and *in vitro* Oxaliplatin induced PROb cell senescence in tumors **(A-B)**. Tumor-bearing rats were treated by oxalipatin or saline solution (control) at day 21. (A) X-Gal staining (blue) of senescent cells in tumors at day 29 (black bar, 100μm). (B) Staining of tumor cells (anti-cytokeratin Ab, blue) and senescent cells (DDAO, red) in tumors at day 29. Images are representative of 3 independent experiments with 3 rats per group. Oxaliplatin induced tumor cell senescence in vitro **(C-D)**. PROb or CT26 cells were treated for 5 days with oxaliplatin. (C) Senescent cells in culture were visualized by X-Gal staining (blue). (D) Quantification of DDAO^+^ cells by flow cytometry 8 days after treatment by oxaliplatin. Bars are means ± SEM Significant differences were determined by Mann-Whitney U test. *p < 0.05, **p < 0.01.

**Figure 4:**
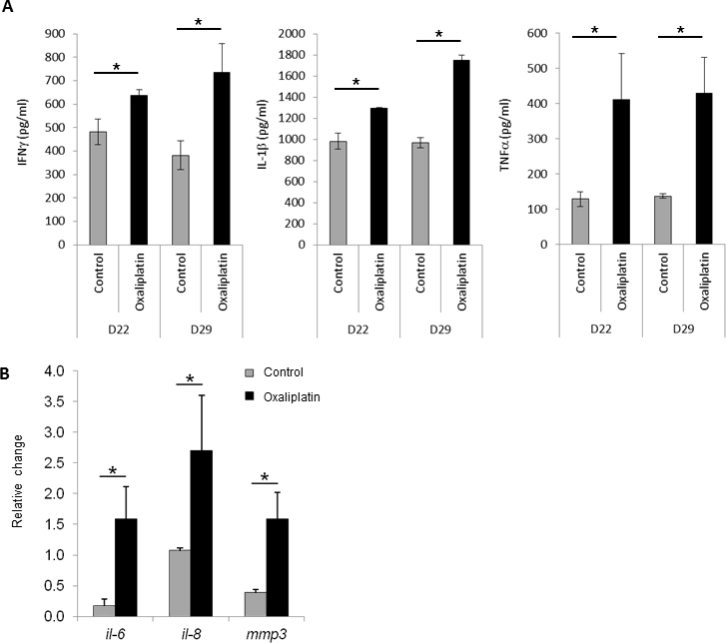
Expression of senescence-associated genes after oxaliplatin treatment *in vivo* and *in vitro* Tumor bearing rats were treated with oxaliplatin or saline solution (control) at day 21. **(A)** At days 22 or 29, tumors were harvested and IFN-γ, IL-1β and TNF-α concentrations were determined by ELISA. **(B)** Ninety six hours later injection, tumors were harvested and expression level of *il-6*, *il-8* and *mmp3* mRNAs was determined by RT-PCR and normalized to *gapdh* gene expression. Bars are means ± SEM. Significant differences were determined by Mann-Whitney U test. *p < 0.05.

We also examined the ability of lipid A and oxaliplatin to induce senescence in the rat PROb and mouse CT26 colon cancer cells. The senescence-associated β-galactosidase (SA-β-Gal) activity in cells was determined by cytochemical detection using the X-Gal substrate or by fluorescence detection using the DDAOG substrate [13]. Lipid A did not trigger senescence either in PROb (Fig 3A) or in CT26 (data not shown) tumors. However, oxaliplatin induced senescence into the PROb cells *in vivo* (Figure 3A and B) and in the two cell lines, PROb and CT26, *in vitro* (Figure 3 C and D). Results obtained with X-Gal staining (Figure 3 A and C) were confirmed with DDAOG staining, another method to detect cellular senescence, visualized by epifluorescence microscopy (Figure 3B) or by flow cytometry analysis (Figure 3D). It is worthy to note that senescence in tumors occurred in cancer cells as some epithelial cells (cytokeratin positive cells) became senescent after oxaliplatin treatment (Figure 3B). Oxaliplatin-mediated cellular senescence was also characterized by the analysis of senescent-associated gene expression (SASP). As shown in Figure 4A, in rats bearing PROb tumors, oxaliplatin induced the expression, at the protein level, of interferon gamma (IFNγ), interleukin(IL)-1β and tumor necrosis factor (TNF) α (Figure 4A). This expression occurred 24 h after treatment and persisted at least 9 days. Some other SASP have been induced by oxaliplatin at the level of mRNA such as IL6, IL8 and matrix metalloproteinase (MMP) 3 (Figure 4B). These results indicate that oxaliplatin but not lipid A is a potent inducer of senescence *in vivo*.

### Oxaliplatin induced chemokines production and recruitment of N1 tumor-associated neutrophils

**Figure 5:**
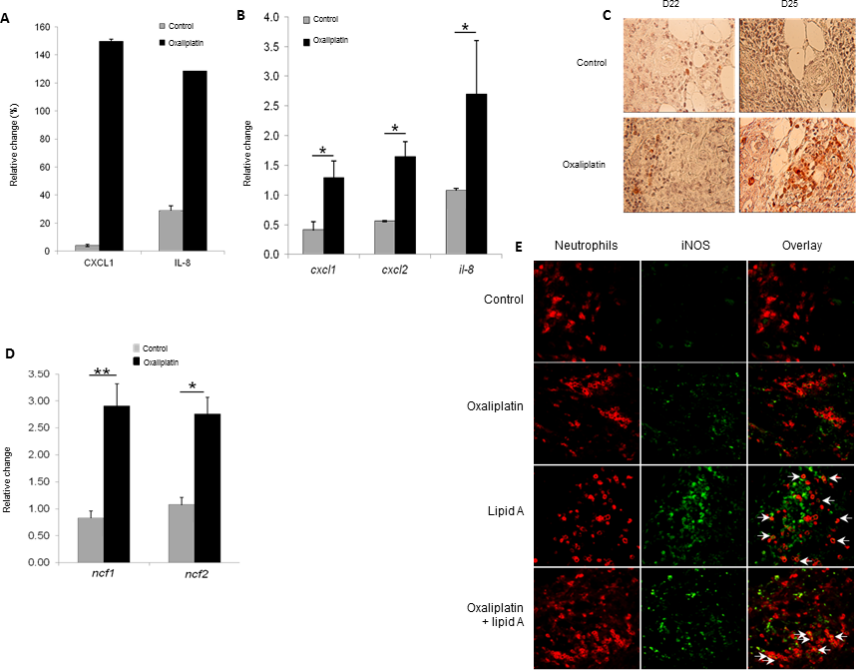
Oxaliplatin induced chemokine production which associated with lipid A, induced the iNOS-expressing-neutrophil recruitment **(A)** Eight days after injection of oxaliplatin or saline solution (control), blood was collected and plasma level of chemokines was analysed by cytokine array. The relative abundance of CXCL1 and IL-8 was expressed as a percentage of an internal control. **(B)** Tumor-bearing rats were treated by oxaliplatin or saline solution (control) at day 21. Ninety six hours later, tumors were harvested and expression levels of *cxcl1*, *cxcl2* and *Il-8* mRNAs were determined by RT-PCR and normalized to *gapdh* gene expression. **(C)** The recruitment of neutrophils in tumors from control or oxaliplatin-treated rats was determined by immunostaining (anti-HIS48 Ab, red) of tumors harvested at day 22 and 25. **(D)** As in **(B)**, tumors were harvested and RNA was extracted. Expression levels of *ncf1* and *ncf2* were determined by RT-PCR and normalized to *gapdh* gene expression. Bars are means ± SEM. Significant differences were determined by Mann-Whitney U test. *p < 0.05, **p < 0.01. **(E)** Tumors from control, oxaliplatin, lipid A or oxaliplatin + lipid A rats were removed at day 29, fixed, cut into 5-mm cryosections and stained for neutrophil (anti-HIS48 Ab, red) and iNOS (anti-iNOS Ab, green). Merged signals (yellow) are pointed with white arrows in the overlay pictures. **(C** and **E)** are representatives of 3 independent experiments with 3 animals per group (scale bars = 50 μm).

Since senescence has been often associated with pro-inflammatory status, we analyzed the ability of oxaliplatin to induce an increased expression of cytokines in blood of tumor-bearing rats. Using a cytokine array, we determined that treatment of rats with oxaliplatin for eight days induced the high expression of CXCL1 and IL-8, two chemokines involved in the recruitment of some immune cells, particularly the neutrophils (Figure 5A). Determination of neutrophil-specific chemokines expression by RT-PCR confirmed that oxaliplatin increased *cxcl1*, *cxcl2* and *il-8* gene expression in colon tumors (Figure 5B). Immunohistostaining techniques and immunohistofluorescence showed that treatment of rats with oxaliplatin for 1 to 4 days triggered the recruitment of neutrophils into tumor microenvironment as attested by the expression of HIS-48, a specific plasma membrane antigen of rat neutrophils (Figure 5C). Neutrophils recruitment was confirmed by the increased expression of neutrophil cytolytic factor 1 (ncf1) and 2 (ncf2), two components of phagocyte NADPH oxidase expressed by neutrophils (Figure 5D). The density of neutrophils was higher inside the tumors from rats treated with the combination oxaliplatin and lipid A than from rats treated with oxaliplatin or lipid A alone (Figure 5E). The neutrophil infiltration is maintained during the lipid A treatment, at least until day 43 (data not shown). Interestingly, lipid A but not oxaliplatin induced the expression of inducible nitric oxide synthase (iNOS) in a fraction of tumor-associated neutrophils (Figure 5E). These results indicated that oxaliplatin induced the recruitment of neutrophils into tumors through the induction of neutrophil specific chemokines. In addition, a part of these neutrophils exhibited a lipid A-dependent expression of an antitumor neutrophil phenotype (N1) that may participate to the antitumor efficacy of oxaliplatin and lipid A. Neutrophils were not the only leucocytes that infiltrated tumor microenvironment. We also observed the recruitment of macrophages and lymphoid cells but at a less extend than neutrophils and occurred later in the case of lymphocytes, 9 days after oxaliplatin treatment (data not shown).

## DISCUSSION

The life span of rats bearing large nodules of colon cancer was greatly increased when animals were treated with the platinum derivative drugs cisplatin or oxaliplatin, together with an analog of lipid A. When treated animals were submitted to autopsy, most of them were free of nodules. This observation demonstrates that the treatment induced the regression of large peritoneal tumor nodules. Indeed, peritoneal carcinomatosis on the one hand and large colon cancer nodules on the other hand are associated with poor clinical outcome [14]. Thus the antitumor efficacy of these drugs combination arises as a new, promising strategy in the fight against cancer progression. In earlier studies, this lipid A analog was shown to induce tumor regression and the recovery of rats bearing macroscopic peritoneal carcinomatosis of colon cancer when treatment started on day 14 post-cell inoculation. It was shown that the treatment with lipid A analog alone induced iNOS expression and tumor cell apoptosis within 24 hours [9, 11, 15]. Here, we show that the antitumor efficacy of the lipid A analog alone was strongly decreased when the treatment started later, *i.e.* on day 28 post-cell inoculation (Figure 1). However, lipid A maintained its ability to induce tumor cell apoptosis in large tumors sensitized by platinum derivatives (Figure 2). It has been reported for some cancers that TLR4 agonists represent promising vaccine adjuvants on the one hand and are successfully and safely used in classical monotherapy on the other hand. Indeed, clinical benefit was observed in a randomized Phase III trial after vaccinating CRC patients with autologous tumor cells and BCG as adjuvant [16]. Monophosphoryl lipid A is also used as vaccine adjuvant with excellent safety and immunogenicity in several cancers [17, 18]. Additional TLR2/TLR4 agonists such as picibanil have been shown to be well tolerated and effective as an adjuvant to cisplatin in a cohort of patients with malignant pleural effusions [19]. Moreover, our team also performed a phase I clinical trial with the synthetic lipid A OM-174 in which its safety and tolerance was demonstrated in patients suffering from cancer [12].

Our results also showed that although lipid A induced cancer cell apoptosis, it failed to increase cellular senescence, contrarily to oxaliplatin which did not act on apoptosis but was a potent inducer of cellular senescence. Even though we did not demonstrate the involvement of apoptosis and senescence in tumor regression, one can speculate that these two pathways should contribute to the antitumor efficacy of oxaliplatin and lipid A, since abundant evidence points to a crucial physiological role for apoptosis and senescence in counteracting tumorigenesis. Recent studies reported that chemotherapy-induced senescence is one of key determinants of tumor response to therapy. For example, cisplatin has been demonstrated to induce cellular senescence in various cancer cells [20, 21]. Oxaliplatin also induced cell senescence in hepatocellular carcinoma cell lines [22]. To our knowledge, there are no data in the literature about the ability of oxaliplatin to induce senescence in colon cancer cells. It was recently reported that 5-FU/leucovorin-treated CRC patients achieved a significantly longer progression free survival when presenting with senescence index-positive tumors before treatment [23]. Based on these data, we can speculate that CRC patients with positive senescence index may represent a cohort of patients with a favorable outcome when treated with lipid A or conversely that CRC patients with negative senescence index may represent good candidates for oxaliplatin/lipid A treatment.

Even though we did not analyze the molecular mechanisms by which oxaliplatin induced cellular senescence, several mechanisms have been reported in the literature. Recent evidence emerges that the mammalian target of rapamycine (mTOR) pathway is involved in cellular senescence [24]. Indeed, inhibition of this pathway, for example, by hypoxia [25], by contact inhibition or by high cell density [26] suppresses senescence program. Further, reactivation of mTOR in quiescent cells causes senescence after treatment with DNA damaging agents [27]. In our case, mTOR activation could be at the origin of oxaliplatin-mediated cellular senescence. Recently, it has been reported that treatment of cholangiocarcinoma cells with oxaliplatin increased the phosphorylation of mTOR [28].

Our results also showed that the antitumor efficacy of oxaliplatin and lipid A was correlated to tumor infiltration of neutrophils. This recruitment within tumors could be due to the induction of cellular senescence by oxaliplatin. Accordingly, it has been observed infiltration of neutrophils, macrophages and NK cells in regressing tumor triggered by p53 activation and thus induction of cellular senescence [29]. Some of these neutrophils expressed iNOS, one hallmark of N1 phenotype of neutrophils. These cells in tumor may have differential states of activation/differentiation, thus taking an antitumorigenic (“N1”) versus a protumorigenic (“N2′’) phenotype [30]. Interestingly, the acquisition of the N1 phenotype after lipid A treatment was also found in neutrophils from bone marrow and spleen (data not shown). Thus, our data show that activation with lipid A could promote the conversion from tumor-promoting to tumor-suppressing neutrophils which could participate to the antitumor efficacy of oxaliplatin and lipid A.

In summary this study describes a new therapeutical strategy to eradicate advanced colon cancers by combining chemotherapeutic agents such as platinum derivatives with immunomodulators such as lipid A analogs. This association is linked with the induction of senescence and apoptosis of tumor cells *in vivo* and with the recruitment of neutrophils with an N1 phenotype.

## METHODS

### Cell culture

PROb colon cancer cells were harvested from a colon carcinoma induced in BD-IX rat [31]. CT26 murine colon cancer cells were obtained from the ATCC. PROb and CT26 cells were maintained at 37°C in HAM's F10 and RPMI medium, respectively, supplemented with 10% FBS (Lonza).

### Animal tumor models and treatments

Male BD-IX rats (2–3 month-old) were purchased from Charles River (L'arbresle, France). Carcinomatosis in BD-IX rats was induced by *i.p.* injection of 1.10^6^ colon cancer PROb cells as previously described [9]. Oxaliplatin or cisplatin treatment (3mg/kg, single *i.p.* injection) was performed 21 days later; control treatment consisted of saline injection. Immunotherapy treatment began 28 days after carcinomatosis induction and consisted of 15 injections of lipid A (1 mg/kg, *i.v.* injections) or of saline solution for controls over a 5-week period. Carcinomatosis in Balb/C mice was induced by i.p. injection of 5.10^5^ CT26 cells. Oxaliplatin treatment (3mg/kg, single i.p. injection) was performed 5 days later; control treatment consisted of saline injection. Immunotherapy treatment began 10 days after carcinomatosis induction and consisted of 5 injections of lipid A (8 mg/kg, i.v. injections) or of saline solution for controls over a 20-day period. All animal procedures were approved by the Ethics Committee of the University of Burgundy (protocols 2006 and 3904).

### Antibodies and reagents

Mouse anti-HIS48 antibody (Ab) (Santa Cruz) was used to characterize tumor-infiltrating rat neutrophils. Tumor cells were identified using a mouse anti-cytokeratin antibody (DakoCytomation). The following Abs were also used: a rabbit anti-iNOS Ab (BD Transduction Laboratories) and a mouse anti-M30 fragment (Roche). Secondary anti-mouse or anti-rabbit Alexa Fluor 568 or 488 conjugates antibodies were purchased from Molecular Probes, Invitrogen. X-Gal (Roche) and DDAOG (Life Technologies) were used to characterize Senescence-Associated-β-Galactosidase (SA-β-Gal) activity into the cells. The lipid A analog OM-174 was kindly provided by OM Pharma (Meyrin, Switzerland).

### Detection of cytokines by Ab array

Blood was collected by heart puncture under isoflurane anesthesia. The same volume of plasma was applied onto antibody-coated membranes for rat cytokine array (R&D Systems) according to the manufacturer's protocol. Arrays were scanned as digital images and signal intensity was quantified with ImageJ software.

### Detection of cytokines by ELISA

IFN-γ, Il-1β and TNF-α concentrations in tumor lysates were detected by ELISA (Uscn Life Science Inc) according to the manufacturer's protocol. Absorbance was read on a spectrophotometer (Asys UVM 340) at 450 nm wavelength.

### RT-PCR

Total RNA was extracted from tumors and cell lines using the TRIzol reagent (Gibco-BRL) according to the manufacter's instructions. RT-PCRs were performed with the Qiagen OneStep RT-PCR kit (Qiagen) according to the manufacturer's protocol. The rat primers used (Invitrogen) were the following: *cxcl1* (f) 5′-GAGAAAGAAGATAGATTGCACCGATG-3′ and (r) 5′-TTCTTCCCGCTCAACACCTTC-3′; *cxcl2* (f) 5′-GTGACACTGAAGAGTTACGATGTCAG-3′ and (r) 5′- CCTGAGGCTCCATAAATGAAAGA-3′; *ncf1* (f) 5′-CACCGAGATCTACGAGTTCC-3′ and (r) 5′-TCCCATGAGGCTGTTGAAGTAC-3′; *ncf2* (f) 5′-GAAAGCATGAAGGATGCCTGG-3′ and (r) 5′- ATAGCACCAAGATCACATCTCCTTCC-3′; *il-6* (f) 5′- TTCTCTCCGCAAGAGACTTCCAGCC-3′ and (r) 5′- AAACGGAACTCCAGAAGACCAGAGC-3′; *il-8* (f) 5′- AGCGGTTCCATCTCGCCATTCATGC-3′ and (r) 5′- TGAACACTGGCCGTTCTTTCCACTGC-3′; *mmp3* (f) 5′- GTACGGCTGTGTGCTCATCCTACC-3′ and (r) 5′- AAGTCTCCATGTTCTTCAACTGCAAAGG-3′. To standardize the cDNA samples, expression of the housekeeping gene *gapdh* was tested with primer pair (f) 5′-GGCACAGTCAAGGCTGAGAATG-3′ and (r) 5′-ATGGTGGTGAAGACGCCAGTA-3′. The PCR products were stained with ethidium bromide (Invitrogen) and analyzed by gel electrophoresis. All densitometry analyses were performed using ImageJ software. Signal intensities were normalized to *gapdh* used as housekeeping gene.

### Histological analysis

Paraffin-embedded samples were cut into 5 μm thick sections. Sections were deparaffined, rehydrated before antigen retrieval by 20-min incubation at 96°C in citrate buffer at pH 7.3 and nonspecific sites were blocked with 5% BSA. For immunohistochemistry (IHC), endogenous peroxidase was inhibited by incubation with 3% H_2_O_2_, the slides were then successively incubated with primary antibodies, biotin-conjugated secondary antibodies and HRP-conjugated streptavidin in PBS with 0.1% Tween and 1% BSA before incubation with AEC solution (Vector Laboratories) and counterstaining with hematoxylin. Apoptotic cells were detected by TUNEL assay using the ApopTag plus Peroxidase In Situ Apoptosis Detection Kit (Millipore). For immunohistofluorescence (IHF), the slides were successively incubated with primary antibodies and fluorochrome-conjugated secondary antibodies in PBS with 0.1% Tween and 1% BSA and were finally mounted using Prolong Gold (Invitrogen). IHC and IHF images were captured on a Nikon Eclipse E400 microscope at a 40x magnification in the visible or fluorescence mode, respectively.

### X-Gal staining

Tumors were fixed in PBS with 20% sucrose and embedded in OCT (Labonord). Samples were cut into 5-μm thick sections and fixed with 2% PFA before incubation with a 1 mg/mL X-Gal solution (Roche) [13] for 24h. Slides were then washed with PBS. Cells fixed with 2% PFA were incubated with a 1 mg/mL X-Gal solution for 24h and washed with PBS. Images were captured on a Zeiss primovert microscope with an AxioCam at 40X magnification.

### DDAOG staining

Tumors were fixed in PBS with 20% sucrose and embedded in OCT (Labonord). Samples were cut into 5-μm thick sections and fixed with 2% PFA before incubation with a 10 μM DDAOG solution (LifeTechnologies) for 60 minutes. Slides were then washed with PBS. Images were captured on a Nikon Eclipse E400 epifluorescent microscope at 40x magnification. Cells fixed with 2% PFA were incubated with a 10 μM DDAOG solution for 60 minutes and washed with PBS before flow cytometry analysis (FACSCalibur, BD Biosciences).

### Statistical analyzes

Experimental data are expressed as means ± SEM. Statistical analysis was performed using R software. The Mann-Whitney *U* test was used to compare data between two treatment groups. Differences were considered statistically significant as follows: *P ≤ 0.05 and **P ≤ 0.01.
